# Optimizing the process of nucleofection for professional antigen presenting cells

**DOI:** 10.1186/s13104-015-1446-8

**Published:** 2015-09-24

**Authors:** Christina Susanne Mullins, Tabea Wegner, Ernst Klar, Carl-Friedrich Classen, Michael Linnebacher

**Affiliations:** Molecular Oncology and Immunotherapy, Department of General Surgery, University Hospital Rostock, Schillingallee 35, 18057 Rostock, Germany; University Children’s Hospital Rostock, Ernst-Heydemann-Str. 8, 18057 Rostock, Germany

**Keywords:** Antigen presenting cells, Dendritic cells, B cells, Nucleofection, Electroporation, IVT mRNA, Technical optimization

## Abstract

**Background:**

In times of rapidly increasing numbers of immunological approaches entering the clinics, antigen delivery becomes a pivotal process. The genuine way of rendering antigen presenting cells (APC) antigen specific, largely influences the outcome of the immune response. Short peptides bear the demerit of HLA restriction, whereas the proper way of delivery for long peptide sequences is currently a matter of debate. Electroporation is a reliable method for antigen delivery, especially using nucleic acids. The nucleofection process is based on this approach with the twist of further ensuring delivery also into the nucleus. Beside the form of antigen, the type of APC used for immune response induction may be crucial. Dendritic cells (DC) are by far the most commonly used APC; however B cells have entered this field as well and have gained wide acceptance.

**Results:**

In this study, we compared B cells to DC with regard to nucleofection efficiency and intensity of resulting antigen expression. APC were transfected either with plasmid DNA containing the reporter gene green fluorescent protein (GFP) or directly with in vitro-transcribed (IVT) GPF mRNA as a surrogate antigen. Out of nearly 100 different nucleofection programs tested, the top five for each cell type were identified and validated using cells from cancer patients. Flow cytometric analyses of transfected cells determining GFP expression and viability revealed a reverse correlation of efficiency and viability. Finally, donor dependant variances were analyzed.

**Conclusion:**

In summary, nucleofection of both DC and B cells is feasible with plasmid DNA and IVT mRNA. And no differences with regard to nucleofectability were observed between the two cell types. Using IVT mRNA omits the danger of genomic integration and plasmid DNA constructs permit a more potent and longer lasting antigen expression.

**Electronic supplementary material:**

The online version of this article (doi:10.1186/s13104-015-1446-8) contains supplementary material, which is available to authorized users.

## Background

The proper presentation of antigens is a crucial step in the orchestration of immune responses. In this context, antigen presenting cells (APC) take up antigens, process them and present epitopes in the respective major histocompatibility complex (MHC) molecules [1]. Generally, two cell types are recognized as so called professional APC: the more prominent representatives are dendritic cells (DC) frequently utilized in all sorts of cellular immunotherapies [2, 3]. However, B cells have also gained wide acceptance as APC [4–7]. Different methodologies have been successfully developed to render these antigen presenters most potently antigen-specific. The simplest approach is to exogenously load peptides onto the MHC molecules. Major drawbacks hereby are restriction to selected MHC molecules and lack of (endogenous) antigen procession [8, 9]. More refined approaches use nucleic acids as source of antigen. With regard to the nucleic acids, DNA is easier to handle than RNA: cloning DNA sequences into respective eukaryotic expression vectors is longstanding routine, and manipulation of cells by viral transduction is commonly performed [10–13]. DNA can be easily amplified by PCR approaches and, as opposed to the peptide synthesis, allows simple and fast testing and optimization of responses to different antigens also in preclinical research laboratories. Since nucleic acids can easily be designed to be recognized by the cellular protein synthesis machinery, they will subsequently be properly translated into long peptides or proteins [14]. Then, the latter will also be substrates of the endogenous antigen processing machinery, resulting in efficient presentation on the cells’ MHC repertoire, thus overcoming the MHC restriction issue [15]. On the downside, manipulation with DNA may lead to stable integration and potentially transgenic cells are a red flag for subsequent clinical applications [16]. RNA may be synthesized from in vitro transcription (IVT) constructs, and so far, no risk of genomic integration has been recognized [17]; au contraire it is considered safe for clinical approaches [18]. In this line of argumentation, the method applied for APC transformation is of importance. Viral transductions bear the risk of stably creating transgenic cells [19, 20]. Thus, non-viral delivery methods are of great interest. Nucleofection is such a technique; by combining standard electroporation with special carrier and buffer solutions, it ensures direct nuclear delivery of the constructs [21].

In this study, we wanted to compare the two major APC types and test nucleofectability using plasmid DNA and IVT mRNA samples. The goal was to optimize the nucleofection process using cells of healthy volunteers and validating the results with—clinically relevant—patient-derived cells.

## Results and discussion

Non-viral delivery of nucleic acids to APC is necessary in many clinical settings; especially in the context of cellular immunotherapies. We optimized the process of nucleofection, a method—which is applicable both for plasmid DNA and IVT mRNA.

### Nucleofection of DC

**Fig. 1:**
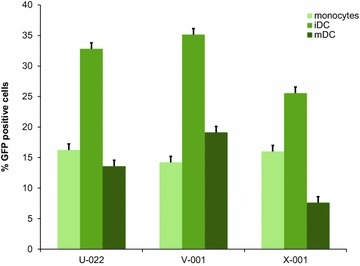
Comparison of nucleofection for monocytes, immature and mature DC. The figure depicts the percentage of viable GPF positive cells post DNA plasmid nucleofection of monocytes, immature (iDC) and mature DC (mDC). The *bars* represent the average percentage (+standard deviation) for two donors and three programs (U-022, V-001 and X-001)

**Fig. 2:**
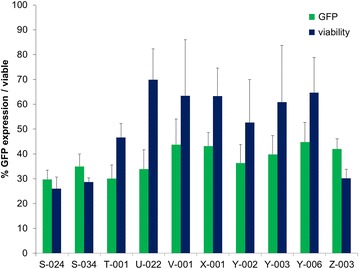
Top 10 DC nucleofection programs. The percentage of GFP positive cells (*green*) as well as viability (*blue*) of immature DC with plasmid DNA for the ten most potent nucleofection programs are represented, the average percentage (+standard deviation) for three donors is given

In a first step, we assessed the differentiation stage at which nucleofection of monocyte derived DC would best be applied. Therefore, we analyzed the nucleofection efficacy—as measured by green fluorescent protein (GFP) positive cells post nucleofection with pmaxGFP plasmid—in monocytes, immature and mature DC (Fig. 1). Nucleofection was most efficient in immature DC with all three programs tested (U-022, V-001 and X001) and viability tended to be highest in immature DC as well (Fig. 1; Additional file 1: Figure S1). In a next step, nucleofection of immature DC with 98 different programs was performed to determine optimal settings (see Additional file 2: Table S1 for a detailed list). The percentage of vital GFP positive cells with the top ten programs ranged from 30 % (±4) to 45 % (±8) with a mean viability rate of 51 % (Fig. 2).

### Nucleofection of B cells (plasmid DNA)

**Fig. 3:**
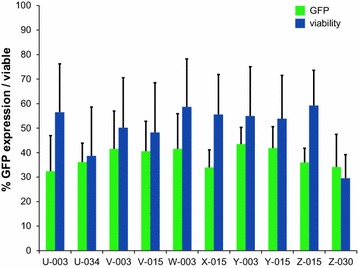
Top 10 B cell nucleofection programs. The percentage of GFP positive cells (*green*) as well as viability (*blue*) of B cells with plasmid DNA for the ten most potent nucleofection programs are represented, the average percentage (+standard deviation) for three donors is given

**Fig. 4:**
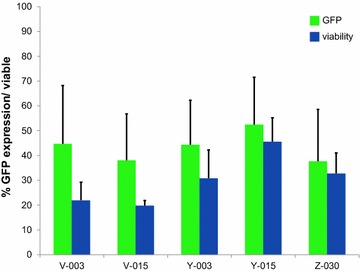
Top 5 B cell nucleofection programs. The percentage of GFP positive cells (*green*) as well as viability (*blue*) of B cells with plasmid DNA for the five most potent nucleofection programs are represented, the average percentage (+standard deviation) for three cancer patients is given

B cells have gained acceptance as APC, so we wanted to compare their nucleofectability to that of DC. A total of 81 programs (see Additional file 2: Table S1, Additional file 3: Table S2 for a detailed list of B cell lines and programs) was tested on an EBV immortalized B cell line (Bc ML) and the ten most effective programs were then verified using two further B cell lines (Bc WR and Bc 736). Nucleofection efficacy was comparable to that achieved with DC; it ranged from 32 % (±15) to 43 % (±7) with a mean viability rate of 53 % (Fig. 3). Since we aimed at transferring the process to patient derived B cell lines, the top five programs were selected taking nucleofection efficiency and viability into account and subsequently tested further on three patient derived B cell lines. Here, the efficacy ranged from 38 % (±19) to 52 % (±19) with a mean viability of 32 % (Fig. 4).

### Nucleofection of B cells (IVT mRNA)

**Fig. 5:**
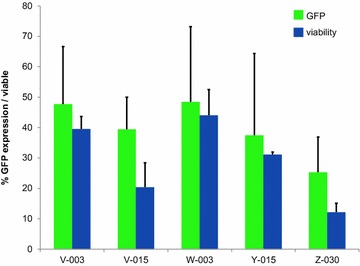
B cell nucleofection with IVT mRNA. The percentage of GFP positive cells (*green*) as well as viability (*blue*) of B cells with IVT mRNA for the five most potent nucleofection programs are represented, the average percentage (+standard deviation) for two cancer patients is given

**Fig. 6:**
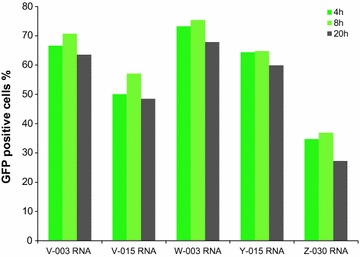
Time kinetic of B cell nucleofection with IVT mRNA. The percentage of GFP positive B cells 4, 8 and 20 h post nucleofection with IVT mRNA using the five most potent nucleofection programs are represented

The risk of stable integration into the host genome makes DNA less favorable with regard to clinical approaches. The nucleofection of B cells using IVT mRNA was thus investigated next. Therefore, two patient derived B cell lines were analyzed using the top five programs as determined with plasmid DNA. The efficacy ranged from 38 % (±27) to 48 % (±25) with a mean viability of 34 % (Fig. 5). Contrary to what is described in literature [22] and thus to some extend surprising, the efficacy was not higher for IVT mRNA compared to plasmid DNA. We thus performed a time kinetics analysis; assessing the percentage of vital GFP positive cells 4, 8, and 20 h post nucleofection (Fig. 6). GFP expression was well detectable already 4 h after nucleofection, peaked at 8 h and started decreasing thereafter but was still detectable 20 h post nucleofection (Fig. 6). Even longer expression periods have been described for IVT mRNA-nucleofected GFP of DC [22, 23]. Although (anti)gene transduction efficiency is by far not the only factor determining the overall antigen presentation capacity of APC, enhanced efficiency of (anti)gene delivery is likely to improve antigen processing and presentation resulting in increased levels of induced immune responses (reviewed by Garg and colleagues in [24]).

**Fig. 7:**
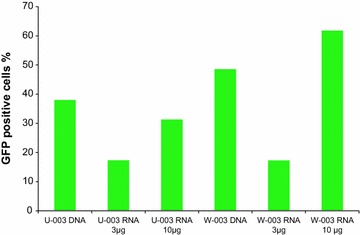
Influence of IVT mRNA quantity on B cell nucleofection. The percentage of GFP positive B cells nucleofected with either 1 µg plasmid DNA, 3 or 10 µg IVT mRNA are depicted for one cancer patient comparing two programs

We finally assessed the influence of the IVT mRNA amount used on the efficacy of nucleofection. We compared the effects using 3 and 10 µg IVT mRNA in addition to 1 µg plasmid DNA nucleofection (Fig. 7). Here, 10 µg IVT mRNA was most effective.

### Comparison of nucleofection with plasmid DNA and IVT mRNA

**Fig. 8:**
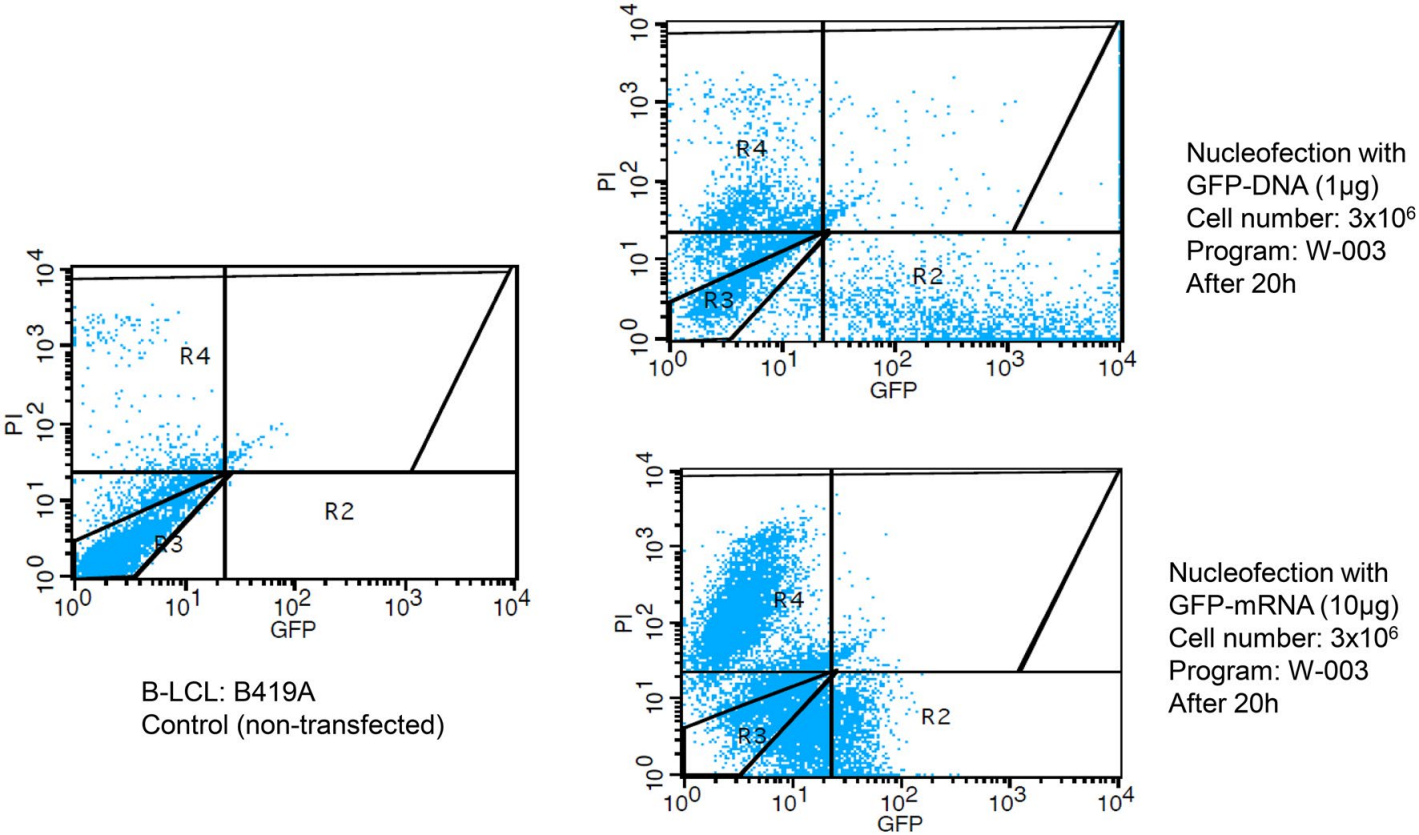
Comparison of B cell nucleofection with plasmid DNA and IVT mRNA. The *dot plots* for flow cytometric analyses of a cancer patient’s B cells post nucleofection with either 1 µg plasmid DNA, 10 µg IVT mRNA or without any nucleic acid (serving as reference) are depicted

The amount of nucleic acid necessary for highly successful nucleofection is tenfold higher for IVT mRNA compared to plasmid DNA (Fig. 7). With regard to the intensity of protein expression—as determined by fluorescence intensity in flow cytometry—plasmid DNA is more potent than IVT mRNA (Fig. 8). Yet, for IVT mRNA an increase in efficacy could be achieved by using more RNA (3 vs. 10 µg); this was not the case for plasmid DNA, where rather a decrease in viability was observed (data not shown). Finally, in terms of viability, no great differences were observed between the two types of nucleic acids (Additional file 4: Figure S2, Additional file 5: Figure S3).

Non viral delivery of nucleic acids to APC using the technology termed nucleofection was feasible and successful for both major types of antigen presenters (DC and B cells) coming from healthy donors as well as from tumor patients. Very recently Maeß and colleagues published an optimized process for nucelofecting of macrophages—a further important player in the immune system. More precisely nucleofection was performed on the human macrophage and monocyte cell line THP-1. Although they only present nucleofection of this cell line with one program, the process was analyzed for plasmid DNA and siRNA [25]. A great variance, in especially with regard to efficacy, between different individuals was observed in our analysis (see SD in Figs. 1, 2, 3, 4, 5). However, the individual most efficient program was among the top five programs selected for the respective cell type. Thus, we do not propose one overall best nucleofection program but rather a handful carefully selected programs to choose amongst.

The nucleofectability of DC and B cells were comparable both with regard to efficacy and handling of cells in the process. Since generation of monocyte derived DC is laborious, expensive and purity as well as efficiency of DC generation differs largely between donors, B cells are favorable in this regard. Besides, they can easily be expanded in vitro which DC cannot [4].

In comparison to B cells from healthy donors, the nucleofection of patient derived B cells was more successful. Yet, the viability was lower. So, the approach is very well applicable in a pathological setting (at least in cancer patients).

Improved transfection efficacy may be achieved, but at the expense of viability. Nevertheless, the nucleofection process should be performed at optimal conditions: minimize time of cells in nucleofection buffer (do not exceed 15 min), pre-heat subsequent culture media (37 °C), prepare material (culture dishes, pipettes, cuvettes) and wash cells out of the cuvette immediately after nucleofection.

Electroporation is an effective (anti)gene transduction method which has in the context of APC not only been proven to allow effective targeting of CD8^+^ T cells [26] but also of CD4^+^ T cells via the MHC class 2 pathway [27].

Finally, we would like to point out that the manner for handling APC prior to and during nucleofection is crucial. Cell densities should not be too high; we recommend splitting B cells, change media and resuspend the cells the day before nucleofection.

## Conclusions

In summary, we here successfully optimized nucleofection of both APC types used in (pre)clinical settings, DC and B cells, for plasmid DNA and IVT mRNA. Subsequent studies profit from our major findings: (1) patient-derived APC are well-suited, (2) due to high individual differences, however, five programs should be tested, (3) DC have to be nucleofected in the immature state, (4) plasmid DNA permits a more potent and longer lasting antigen expression, (5) achievable levels of antigen expression are similar for B cells and DC.

## Methods

### Cell culture

The human colon cancer cell line HROC24, established in our lab [28], was cultured in DMEM/Ham’s F12 (1:1) and all EBV B cell lines (for detailed list see Additional file 3: Table S2) were cultured in IMDM. DC were generated as described before [29]. Monocytes were isolated by MACS cell separation of peripheral blood mononuclear cells using human CD14 MicroBeads (Miltenyi, Bergisch Gladbach, Germany), incubated in RPMI supplemented with Il-4 (20 ng/ml; Immunotools, Friesoythe, Germany) and GM-CSF (1,000 IU/ml; Immunotools) for 5 days and matured using TNFα (120 ng/ml; Immunotools) and Il-1β (120 ng/ml; Immunotools) for two additional days. All culture media were supplemented with 10 % fetal calf serum (FCS Gold, PAA Cölbe, Germany), 2 mmol/l l-glutamine and 1 % penicillin–streptomycin. Cell cultures were incubated at 37 °C with 5 % CO_2_. Media and supplements, if not indicated otherwise, were purchased at Pan Biotech (Aidenbach, Germany). Maturation states of the DC were routinely checked as described before [30].

### Nucleic acids

Nucleofection of plasmid DNA was performed using the pmaxGFP plasmid (Lonza, Basel, Switzerland). For mRNA nucleofection, the GFP gene was cloned from the pCR2.1-EGFP plasmid using EcoRI (Promega, Madison, United States) into the PGEM-3-Z vector (Promega) especially designed for highly efficient IVT. 1 µg NarI (Promega) linearized PGEM-3-Z-GFP plasmid served as template for mRNA synthesis using AmpliScribe T7 Flash, Poly(A) Polymerase Tailing Kit and ScriptCap m7G Capping System reagents by epicentre (Madison, United States) according to the manufacturer’s instructions to produce capped IVT mRNA with a poly(A) tail. All nucleic acid concentration determinations were done using a NanoDrop (Thermo-Scientific, Waltham, United States).

### Nucleofection

Cells were harvested, washed, and resuspended in solution V (Lonza; i.e. 90 mM Na_2_HPO_4_, 90 mM NaH_2_PO_4_, 5 mM KCl, 10 mM MgCl_2_ and 10 mM sodium succinate dissolved in bi-distilled water): DC (1 × 10^4^/µl if not indicated otherwise) or Bc (3 × 10^4^/µl if not indicated otherwise). Subsequently, 100 µl of the cell suspension was mixed with 1 µg plasmid DNA or 10 µg IVT mRNA (if not indicated otherwise), and electroporated in a 0.2 cm cuvette using the Nucleofector™ II device (Lonza). Various nucleofection programs (see Additional file 2: Table S1) were compared in order to assess their effect on transfection efficiency. One million HROC24 cells were nucleofected for each batch IVT mRNA (3 µg) to assure consistent quality.

### Flow cytometry

GFP-transfected cells were checked for GFP expression 20 h (if not indicated otherwise) after nucleofection by flow cytometry. Briefly, cells (5 × 10^5^ cells) were washed once in PBS and resuspended in 200 µl PBS. Propidium iodine at a final concentration of 20 µg/ml was added directly prior to flow cytometric analysis on a FACScalibur analytical flow cytometer (Becton–Dickinson, Heidelberg, Germany) to allow for exclusion of dead cells and thus to simultaneously assess cell viability and GFP positivity. Viability was calculated as follows: 100 % minus  % Propidium iodine positive cells.

### Nucleofection efficiency

The efficiency (defined as the percentage viable-GFP positive cells) for each program and cell line was calculated as follows:

$$100/\left( {{\text{total }} \% \;{\text{viable cells}}} \right) \, \times \, \left( {{\text{total }} \% \;{\text{GFP positive cells}}} \right).$$

## Additional files

10.1186/s13104-015-1446-8 Viability of monocytes, immature and mature DC after nucleofection. The figure depicts the percentage of viable cells post DNA plasmid nucleofection of monocytes, immature (iDC) and mature DC (mDC). The bars represent the average percentage (+ standard deviation) for two donors and three programs (U-022, V-001 and X-001).
10.1186/s13104-015-1446-8 List of programs used for nucleofection.
10.1186/s13104-015-1446-8 List of B cell lines.
10.1186/s13104-015-1446-8 Viability of B cells after nucleofection with plasmid DNA and IVT mRNA. The figure depicts the percentage of viable B cells of a cancer patient post nucleofection with either GFP plasmid DNA or IVT mRNA. The viability for untransfected cells and mock transfected cells are given as reference values.
10.1186/s13104-015-1446-8 Comparison of DNA and IVT mRNA nucleofection of B cells.The figure depicts dot plots for non-transfected B cells (= control) on the left side. The dot plots for DNA (1μgplasmid DNA; upper right) and IVT mRNA (10μg IVT mRNA; lower right) are shown on the right side. B cells(3x106 B419A) were nucleofected using the W-003 program. GFP fluorescence intensity is shown on the Xaxisand PI positivity on the Y-axis.

## Abbreviations

APC: antigen presenting cell
DC: dendritic cell
GFP: green fluorescent protein
IVT: in-vitro transcription
MHC: major histocompatibility complex
