# Supplementary figures and images for: Optimizing the process of nucleofection for professional antigen presenting cells
Source: BMC Res Notes. 2015 Sep 24;8:472. doi: 10.1186/s13104-015-1446-8 (PMC4581479; doi:10.1186/s13104-015-1446-8)

## Slide 1
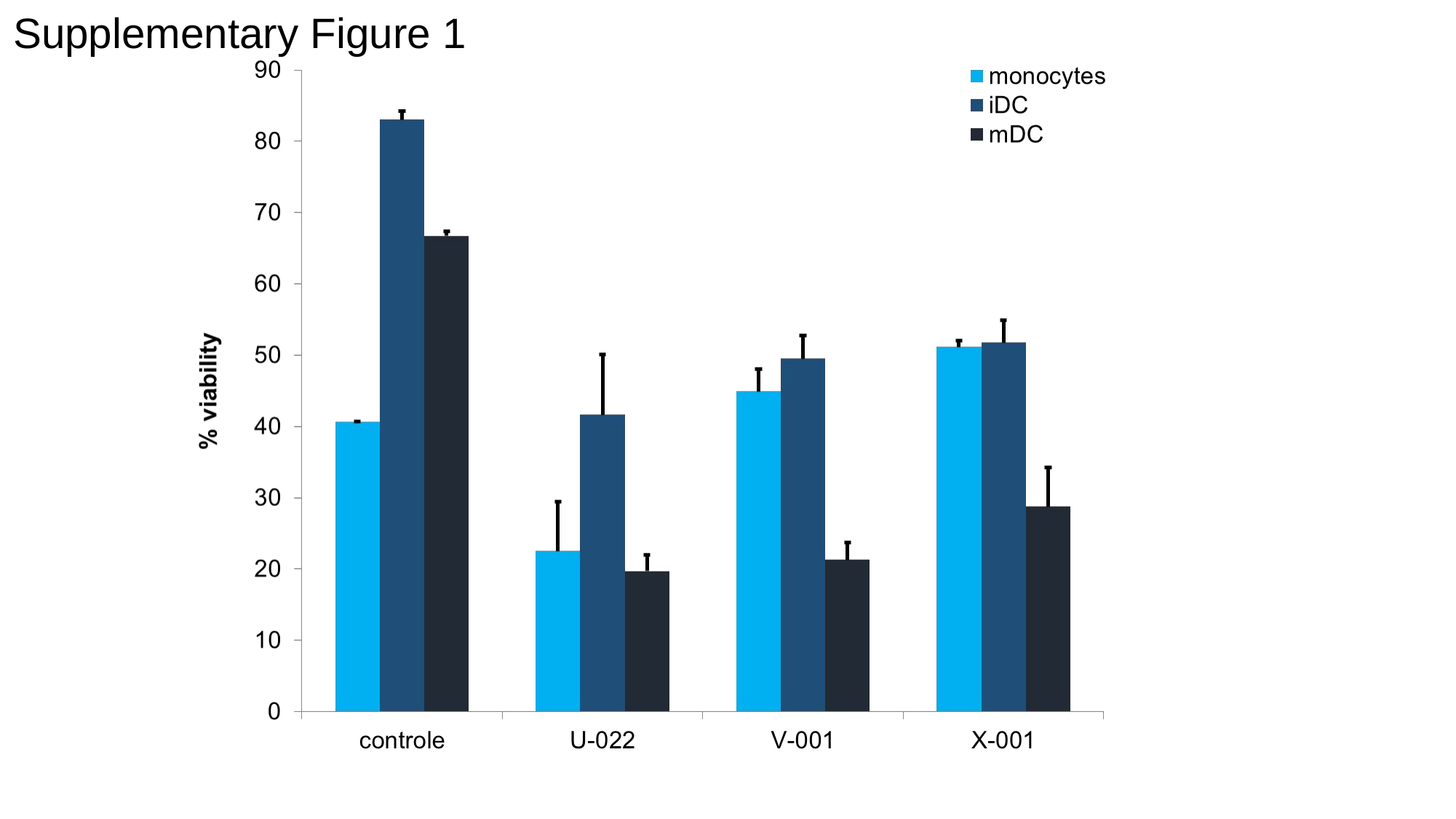

Supplementary Figure 1

Supplement: Supplementary file 1 — 10.1186/s13104-015-1446-8 Viability of monocytes, immature and mature DC after nucleofection. The figure depicts the percentage of viable cells post DNA plasmid nucleofection of monocytes, immature (iDC) and mature DC (mDC). The bars represent the average percentage (+ standard deviation) for two donors and three programs (U-022, V-001 and X-001). [file 13104_2015_1446_MOESM1_ESM.ppt]

## Slide 1
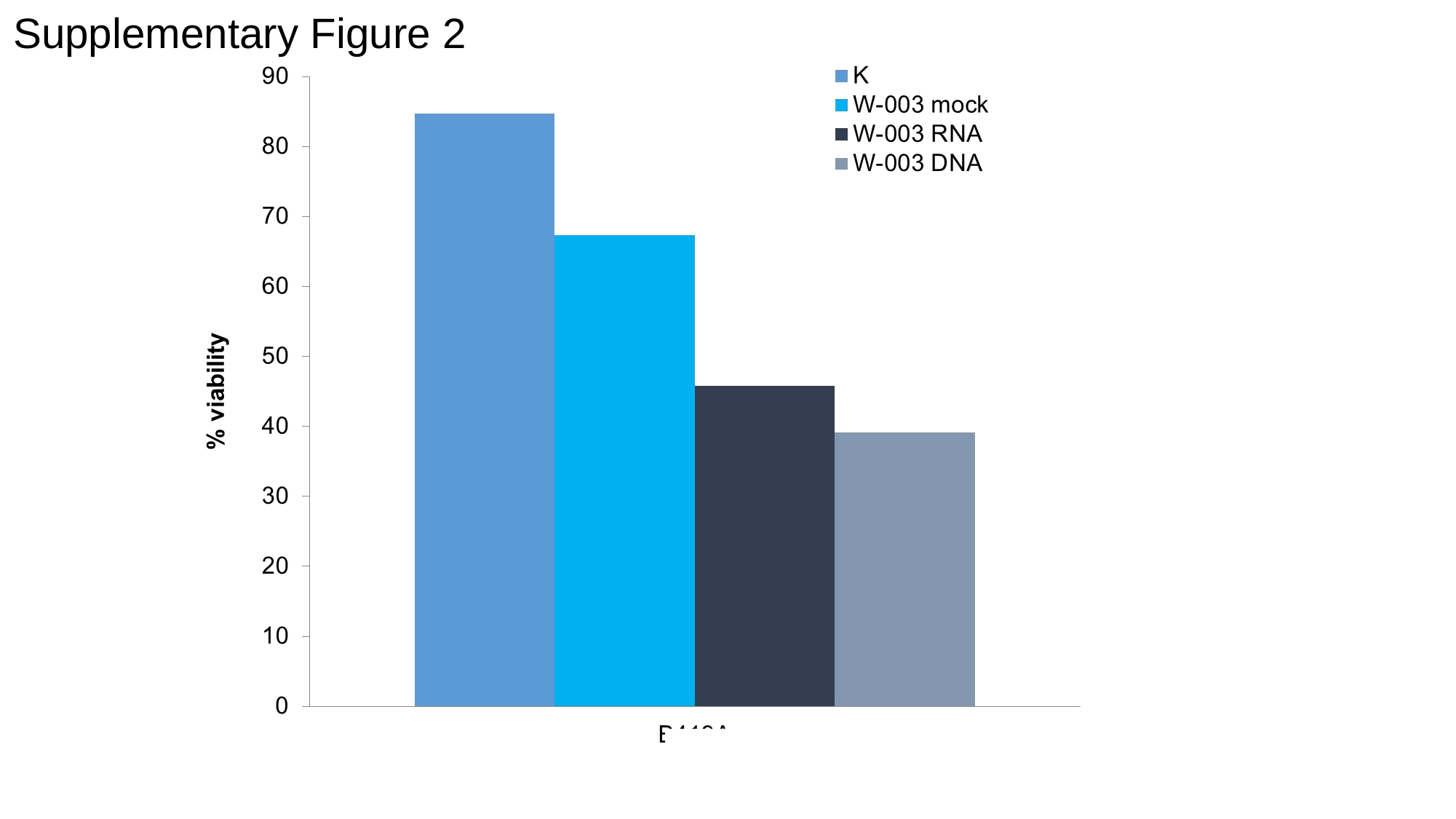

Supplementary Figure 2

Supplement: Supplementary file 4 — 10.1186/s13104-015-1446-8 Viability of B cells after nucleofection with plasmid DNA and IVT mRNA. The figure depicts the percentage of viable B cells of a cancer patient post nucleofection with either GFP plasmid DNA or IVT mRNA. The viability for untransfected cells and mock transfected cells are given as reference values. [file 13104_2015_1446_MOESM4_ESM.ppt]

## Slide 1
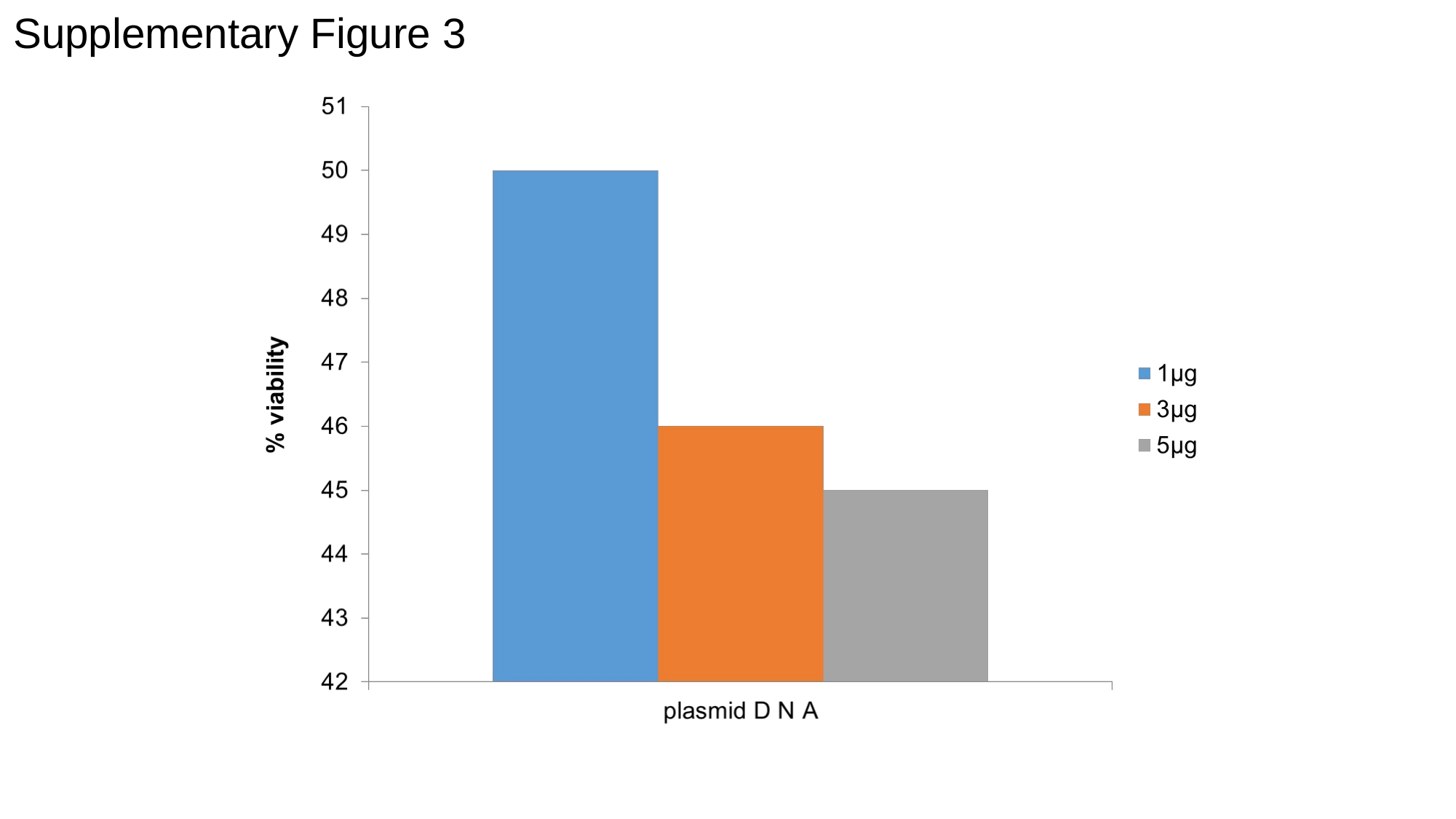

Supplementary Figure 3

Supplement: Supplementary file 5 — 10.1186/s13104-015-1446-8 Comparison of DNA and IVT mRNA nucleofection of B cells.The figure depicts dot plots for non-transfected B cells (= control) on the left side. The dot plots for DNA (1μgplasmid DNA; upper right) and IVT mRNA (10μg IVT mRNA; lower right) are shown on the right side. B cells(3x10^6^ B419A) were nucleofected using the W-003 program. GFP fluorescence intensity is shown on the Xaxisand PI positivity on the Y-axis. [file 13104_2015_1446_MOESM5_ESM.ppt]
