# Supplementary material for: Optimizing the process of nucleofection for professional antigen presenting cells
Source: BMC Res Notes. 2015 Sep 24;8:472. doi: 10.1186/s13104-015-1446-8 (PMC4581479; doi:10.1186/s13104-015-1446-8)
Supplement: Supplementary file 2 — 10.1186/s13104-015-1446-8 List of programs used for nucleofection. [file 13104_2015_1446_MOESM2_ESM.docx]

**Supplementary Table 1: List of programs used for nucleofection**

**DC**

R-001 R-008 R-015 R-024 R-034 S-001

S-008 S-015 S-024 S-034 T-001 T-008

T-015 U-001 U-002 U-003 U-004 U-005

U-006 U-007 U-008 U-009 U-010 U-011

U-012 U-013 U-014 U-015 U-016 U-017

U-018 U-019 U-020 U-021 U-022 U-023

U-024 U-025 U-026 U-028 U-029 U-030

U-031 U-032 U-034 V-001 V-002 V-003

V-004 V-005 V-006 V-007 V-008 V-015

V-024 V-034 W-001 W-002 W-003 W-004

W-005 X-001 X-002 X-003 X-004 X-005

X-006 X-007 X-008 Y-001 Y-002 Y-003

Y-004 Y-005 Y-006 Y-007 Y-008 Y-009

Y-011 Y-013 Y-015 Y-017 Y-019 Y-021

Y-023 Y-025 Y-027 Y-029 Y-031 Y-033

Z-001 Z-002 Z-003 Z-004 Z-005 Z-006

Z-007 Z-008

**B cells**

A-001 A-015 A-034 B-001 B-015 B-034

C-001 C-015 C-034 D-001 D-015 D-034

E-001 E-015 E-030 F-003 F-015 F-030

G-003 G-015 G-030 H-003 H-015 H-030

I-003 I-015 I-030 J-003 J-015 J-030

K-015 K-003 K-030 L-015 L-003 L-030

M-003 M-015 M-030 N-003 N-015 N-030

O-003 O-015 O-030 P-003 P-015 P-030

Q-003 Q-015 Q-030 R-003 R-015 R-030

S-003 S-015 S-030 T-003 T-015 T-030

U-003 U-013 U-015 U-020 U-030 U-034

V-003 V-015 V-030 W-003 W-015 W-030

X-003 X-015 X-030 Y-003 Y-015 Y-030

Z-003 Z-015 Z-030

Full list of nucleofection programs used for DC and B cells transfection.
