# Supplementary material for: Optimizing the process of nucleofection for professional antigen presenting cells
Source: BMC Res Notes. 2015 Sep 24;8:472. doi: 10.1186/s13104-015-1446-8 (PMC4581479; doi:10.1186/s13104-015-1446-8)
Supplement: Supplementary file 3 — 10.1186/s13104-015-1446-8 List of B cell lines. [file 13104_2015_1446_MOESM3_ESM.docx]

**Supplementary Table 2: List of B cell lines**

**Healthy donors** **CRC patients**

Bc ML Bc HROC24

Bc WR Bc HROC87

B736 B419A

Full list of healthy donor and cancer patient-derived B cell lines used in this study.
